# The Influence of Coastal Access on Isotope Variation in Icelandic Arctic Foxes

**DOI:** 10.1371/journal.pone.0032071

**Published:** 2012-03-01

**Authors:** Fredrik Dalerum, Anna Perbro, Rannveig Magnusdottir, Pall Hersteinsson, Anders Angerbjörn

**Affiliations:** 1 Centre for Wildlife Management, University of Pretoria, Pretoria, South Africa; 2 Mammal Research Institute, Department of Zoology and Entomology, University of Pretoria, Pretoria, South Africa; 3 Department of Zoology, Stockholm University, Stockholm, Sweden; 4 Faculty of Life and Environmental Sciences, University of Iceland, Reykjavik, Iceland; University of California, Berkeley, United States of America

## Abstract

To quantify the ecological effects of predator populations, it is important to evaluate how population-level specializations are dictated by intra- versus inter-individual dietary variation. Coastal habitats contain prey from the terrestrial biome, the marine biome and prey confined to the coastal region. Such habitats have therefore been suggested to better support predator populations compared to habitats without coastal access. We used stable isotope data on a small generalist predator, the arctic fox, to infer dietary strategies between adult and juvenile individuals with and without coastal access on Iceland. Our results suggest that foxes in coastal habitats exhibited a broader isotope niche breadth compared to foxes in inland habitats. This broader niche was related to a greater diversity of individual strategies rather than to a uniform increase in individual niche breadth or by individuals retaining their specialization but increasing their niche differentiation. Juveniles in coastal habitats exhibited a narrower isotope niche breadth compared to both adults and juveniles in inland habitats, and juveniles in inland habitats inhabited a lower proportion of their total isotope niche compared to adults and juveniles from coastal habitats. Juveniles in both habitats exhibited lower intra-individual variation compared to adults. Based on these results, we suggest that foxes in both habitats were highly selective with respect to the resources they used to feed offspring, but that foxes in coastal habitats preferentially utilized marine resources for this purpose. We stress that coastal habitats should be regarded as high priority areas for conservation of generalist predators as they appear to offer a wide variety of dietary options that allow for greater flexibility in dietary strategies.

## Introduction

The dietary specialization of predator populations has far reaching consequences for their ecological impacts. In a highly influential study, Roughgarden [1] highlighted that the dietary breadth exhibited by a predator population may depend not only on the specialization of individual predators, but also on dietary overlap between individuals. A population of individually specialized foragers with low dietary overlap will result in a population with a broad niche breadth, similar to a population of individual generalists with high dietary overlap, while a population of individually specialized foragers with high dietary overlap will result in a population with a narrow niche breadth [2]. However, although the theoretical models by Roughgarden [1], [2] assume equal individual specialization within a population, there may also be a mix of individual strategies so that the full dietary breadth of a population is determined also by the variation between individuals in terms of their individual specialization. It is therefore important to evaluate how population-level specializations are dictated by intra- versus inter-individual dietary variation as well as individual variation in individual dietary specialization [3].

Dietary specialization of predators may either be obligatory, due to a lack of alternative prey, or facultative, in which a predator switches to temporarily abundant prey when these are sufficiently common to be the most profitable to prey upon [4]. Dietary breadth is thus typically broader for predator populations in environments with larger prey diversity [5]. For generalist predators, environments with a varied prey base are therefore often more productive and can better sustain predator populations [6]. For terrestrial carnivores, coastal habitats usually provide high prey diversity with prey from both the terrestrial and the marine biome as well as typically coastal prey. Many species of terrestrial carnivores utilize this diversity for feeding, and coastal habitats can sustain higher predator densities than terrestrial regions [7], [8]. However, to what extent such an expanded foraging niche in costal habitats is caused by a diversification of the diet of all individuals, by an increased individual niche separation or by an increased range of dietary strategies has so far rarely been tested with empirical data.

The arctic fox (*Vulpes lagopus*) is a medium sized canid with a circumpolar distribution in the northern hemisphere. Two distinct ecological adaptations have been identified. Arctic foxes living in arctic tundra habitat are heavily dependent on microtine rodents [9]–[12]. Foxes from these populations have large maximum litter sizes (>12) but also a large annual variation in breeding effort [13]. In contrast, foxes inhabiting coastal habitats, predominantly in Iceland, Svalbard and western Greenland, exhibit a varied diet with a significant marine influence [14]–[16]. Foxes in these areas typically have smaller litter sizes than tundra-living foxes but breed more regularly [14], [17]. These differences have mainly been attributed to variation between the two habitats in terms of temporal variation in food supply, but also to the predictability of available food resources [13], [18]–[19]. The arctic fox is thus an appealing candidate species for examining if the increased prey diversity in coastal areas results in individual generalists or in individual specialists with low dietary overlap between them. Moreover, since the contrasting resource availability between tundra and coastal habitats has given rise to profound differentiation in reproductive strategies [19], it is also an interesting candidate species for evaluating how individual resource specialization transcends into strategies for rearing offspring.

Analysis of naturally occurring stable isotopes has become an established tool to investigate foraging ecology of many animal species [20], [21]. Provided that individuals have dietary options of contrasting isotope values, isotope niche breadth can be used as a proxy of dietary niche breadth [22], [23]. It has consequently been suggested that stable isotope data can be a potentially powerful tool to examine questions related to dietary specializations within and between individuals [23]–[25]. This technique has been used to examine individual dietary specialization in a wide range of species, from marine [26], [27] and terrestrial [28], [29] predators to small passerine birds [30], exemplifying the utility of the approach. Information of isotope variation within individuals can come from three potential sources [20]. First, repeated samples can be taken of a tissue with relatively (compared to the sample regime) short turnover. Second, tissues with progressive growth, such as hair or feathers, will retain isotope information and a single sample will thus represent a time series of isotope values corresponding to the growth rate of the tissue in question. Third, tissues will contain isotope information specific to its elemental turnover rate. Therefore, comparisons of isotope values between tissues with different metabolic rates can thus also reveal temporal dietary variation within individuals. However, we note that all these options only quantify temporal variations in isotope values within individuals, and do not estimate dietary diversity at any given point in time.

In this study we used stable isotope data from tissues with different metabolic rates to address questions regarding between and within individual variation in isotope niche breadth in arctic foxes from coastal and inland habitats on Iceland. We also investigated if any habitat related variation in niche breadth differed between adults and juveniles. Many animals shift resource use through their life stages [31]. Juvenile arctic foxes rely on food from their parents until they can forage independently, which in Iceland occurs at approximately 4 months [32]. Comparing individual niche breadth between adults and juveniles will therefore render important information on how habitat related variation in resources is utilized not only for individual feeding strategies, but also for variation in strategies of raising offspring. Our study focuses on three main questions: I) do arctic foxes from coastal and inland habitats differ in their isotope niche? II) how do any such habitat related differences in isotope niches compare to individual isotope niche breadth in foxes from each habitat? III) how are habitat related differences both in population and individual isotope niche breadth affected by the life stage of the animal?

## Materials and Methods

### Study area

Iceland (63°20–66°30N; 13°30′–24°30′W) can be divided into two main habitat types, coastal and inland. Despite its latitude, sea-ice rarely freezes around Iceland. Therefore, foxes in coastal habitats typically have access to an ice-free shoreline throughout the year with a seasonally stable availability of food resources. Such food resources come both directly from the ocean in the form of carrion, fish and marine invertebrates and indirectly in the form of seabirds. Inland habitats experience substantial seasonal fluctuations in resource availability. In the absence of resident populations of microtine rodents, the diet of Icelandic arctic foxes without access to the shoreline consists mainly of rock ptarmigan (*Lagopus muta*), waders, geese and passerine birds, as well as sheep carcasses and insects [16].

For the purpose of this study we defined coastal habitat as terrain within 3 km of the shore, while inland habitat was defined as all terrain ≥10 km from the shoreline. The arctic fox is the only canid species living in Iceland and they are culled legally in all seasons, including the denning season.

### Tissue sampling, sample preparation and stable isotope analysis

**Figure 1:**
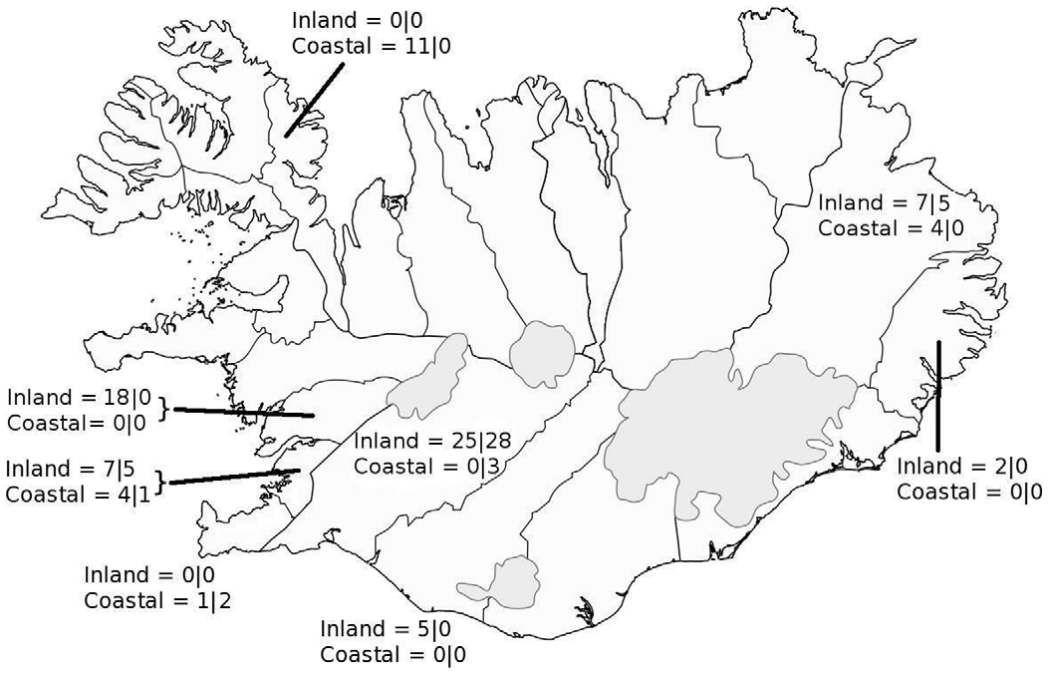
Map of Iceland with the number of arctic foxes (adults|juveniles) that were sampled in coastal (≤3 km from the shore line) and inland (≥10 km from the shore line) habitats in each province. We accounted for potential spatial autocorrelation in isotope values from foxes from the same province by adding it as a random term in statistical models.

We collected fur, muscle and bone samples from legally culled foxes from 8 provinces across Iceland (Figure 1). Foxes were donated to Professor Hersteinsson to be used for research purposes. The majority of the foxes where culled between June 1 and July 7 2003 (70 out of 84 individuals), although we included samples from 5 adult animals culled in April and May 2003 and 4 adult and 5 juveniles from July 2002. We categorized animals as adults or juveniles, with juveniles being offspring still remaining at their natal dens. In addition, we collected a few soft tissue samples from potential prey species. These are not reported as a comprehensive range of available prey species, nor are they intended for quantitative purposes. However, they exemplify the extended isotope niche available to foxes inhabiting coastal habitats compared to inland, which is a fundamental assumption behind using stable isotopes for dietary niche breadth analyses.

We dried muscle samples for 24 hours at 60°C, pulverised them by hand and following Liden et al. [33] removed lipids according to Bligh and Dyer [34]. After lipid extraction, we re-dried the samples before final analysis of isotope ratios. We obtained bone powder from lower jaw bones using a small hand-held electric drill, and extracted collagen with the modified Longing method [35]. We removed lipids from the extracted collagen samples using the same method as for muscle samples. We rinsed hair samples by sonicating them in a chloroform/methanol/water (1∶2∶1) solution to remove surface attached lipids and contaminants.

We conducted analysis of ^13^C/^12^C and ^15^N/^14^N ratios on a Carlo Erba elemental analyzer (E1108 CHNS-O) connected to a Fison Optima isotope ratio mass spectrometer, with a standard deviation of ≤0.1‰. Isotope values are presented as δX values, which represent the proportional deviation in parts per thousand (‰) from a standard:
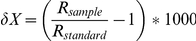
where X is either ^13^C or ^15^N, and R is either ^13^C/^12^C or ^15^N/^14^N, respectively. The accepted standard for carbon is Pee Dee Belemnite (PDB) and the standard for nitrogen is air. Raw isotope data for each tissue, habitat and age class are given in table S1.

### Statistical analyses

We used mixed linear models to test for main and interaction effects of habitat, age of animal and tissue on δ^13^C and δ^15^N values in arctic foxes. In the models, δ^13^C and δ^15^N were used as response variables, and habitat (coastal or inland), age (adult or juvenile) and tissue (fur, muscle and collagen), as well as all interaction effects, were used as fixed effects. We added province and tissue nested within individual as random terms to account for potential spatial autocorrelation as well as non-independence of measurements of different tissues from the same individual. A variance power function was used to account for non-equal variances between factor levels [36]. Since there were no differences between months in δ^13^C (analysis of variance, F_4,72_ = 0.51, p = 0.73) or δ^15^N (analysis of variance, F_4,72_ = 1.14, p = 0.34) of muscle samples from adult individuals, we pooled samples from all months in the analyses.

To estimate total isotope niche breadth in each habitat and for each age category, we calculated the Euclidean distances in a two dimensional isotope space formed by δ^13^C and δ^15^N values. The distances were calculated from each sample to the group centroids of each habitat, age class and tissue (analogous to a multivariate variance decomposition following Anderson [37]). These distances were then used as a response variable in a mixed linear model with the same structure as described above.

To estimate individual isotope niche breadth, we compared δ^13^C and δ^15^N in muscle and collagen within individuals from which we had samples from both tissues. Bone collagen has a very slow turnover, which for long-living species spans several years [38]. Muscle has a substantially faster turnover rate, which for medium sized mammals approximates one month [39]. Although protein turnover rate in mammal bone is substantially higher in juveniles than in adults [40], similar age related differences have been found for protein turnover in vertebrate muscle tissue [41]. We do not know the specific differences between adults and juveniles in turnover rates of collagen and muscle in foxes. However, if both tissues have faster turnover rates in juveniles than in adults, isotope values of the two tissues will reflect shorter time periods in juveniles, but they will still reflect different time periods in relation to each other. Therefore, comparisons of isotope values in tissues with different metabolic rates will probably be less powerful as a measurement of individual niche breadth in juveniles compared to adults, but could still render information regarding individual isotope niche breadth. Each tissue has a tissue specific fractionation rate (i.e. discrimination of heavy vs. light isotope in incorporation into proteins), and unless information of such fractionation rates are available direct comparisons between tissues are not meaningful [20]. However, any contrasts in the difference between tissues are likely to not be biased by fractionation processes. To evaluate differences in within individual isotope variation between adult and juvenile foxes from coastal and inland habitats, we used the Euclidean distance between muscle and collagen within individuals as a response variable in a mixed model with the same effects as described above, but only including province as a random term since only one data point per individual was included in the model.

Since the within individual niche breadth typically is estimated as the average variation within individuals [1]–[2], [42], it does not capture between individual variation in individual niche breadth. There are, however, no a-priori reasons to neglect that individual predators within a single population may adopt contrasting strategies. Therefore, to compare variation between individuals in terms of their individual isotope niche breadth between adult and juvenile foxes from coastal and inland habitats, we calculated the Euclidean distance to group centroids (again for each habitat and age class) in a two dimensional isotope space formed by the differences in δ^13^C and δ^15^N between muscle and collagen. These distances can be regarded as a measure of the dispersion of intra-individual differences between sample groups, and was used as a response variable in a mixed model as described above, with habitat, age and a two way interaction as fixed effects and province as random term.

Finally, Roughgarden [1] suggested that that the ratio of individual niche breadth (Within Individual Component, WIC) to the total niche breadth of a population (Total Niche Width, TNW) can be used as a quantitative index of individual diet specialization. We calculated a two tissue isotope proxy for WIC/TNW. We estimated WIC as the average Euclidean distance from samples to the group centroid within a given individual in a two dimensional isotope space consisting of δ^13^C and δ^15^N. Similarly, we estimated TNW as the average Euclidean distance between each sample to the group centroid of each habitat and age class. We used the ratio WIC/TNW as a predictor in a mixed linear model with the same structure as described above, i.e. with habitat, age and a 2-way interaction as fixed effects and province as random term. Although differences in fractionation between tissues would influence these Euclidean distances, the differences should remain constant for comparisons of within versus between individual distances, and should hence not significantly influence the interpretability of the results.

Newsome et al. [23] advocated transforming the isotope δ space into a p space of dietary proportions using mixed source models [43], [44]. We have refrained from using such models to estimate isotopic contribution from specific dietary sources since they rely on a number of assumptions regarding animal physiology that have not yet been empirically tested [20], [21], and a growing body of literature suggest that system specific experimental data on fractionation values and elemental turnover may be necessary to appropriately interpret results from mixing models [21], [45]–[47].

All statistical analyses were carried out using the statistical software R (version 2.12.1 for Linux, freely available at http://www.r-project.org). Multivariate analyses were carried out using functions in the contributed packages vegan [48] and bio3d [49].

## Results

**Figure 2:**
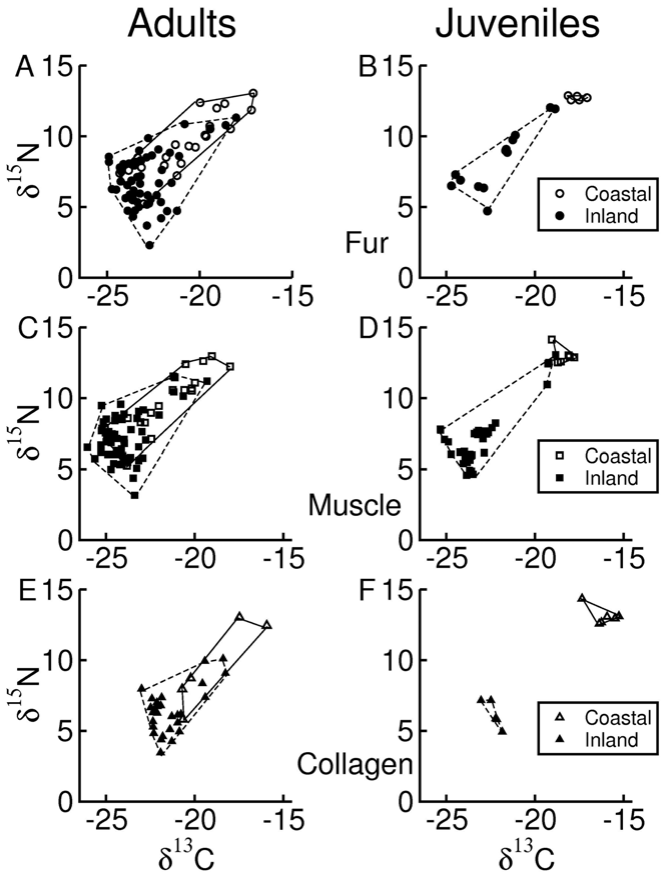
Biplots of δ^13^C and δ^13^N values of fur (A, B), muscle (C, D) and collagen (E, F) samples from adult and juvenile arctic foxes from coastal (open symbols) and inland (closed symbols) habitats on Iceland.

Habitat, age and tissue interacted in their effects on δ^15^N values (F_2, 121_ = 4.01, p = 0.02) but not in their effects on δ^13^C (F_2, 121_ = 0.84, p = 0.43) (Table 1). For δ^13^C, we instead found significant interaction effects of habitat and age (F_1, 124_ = 14.9, p<0.01) and of habitat and tissue (F_2, 121_ = 3.33, p = 0.04). Both adult and juvenile coastal foxes were enriched in ^13^C as well as in ^15^N compared to inland foxes (Figure 2a–f), and muscle and fur samples from adult coastal foxes were depleted in both ^13^C and ^15^N compared to samples from juvenile coastal foxes. Similarly, muscle from adult inland foxes was depleted in ^13^C compared to juveniles. As predicted, prey available only in coastal habitats were enriched in both ^13^C and ^15^N compared to prey available also in inland habitats (Table 2), with a resulting broader isotope niche available for foxes feeding in coastal habitats.

**Table 1:** Results from linear mixed models on the effects of habitat (coastal or inland), age of animal (adult or juvenile) and tissue (fur, muscle and collagen) on δ^13^C and δ^15^N in Icelandic arctic foxes.

Fixed effect	*DF*	*F*	*P*
δ^13^C			
Habitat	1, 124	101.68	<0.001
Age	1, 124	4.48	0.036
Tissue	2, 121	168.90	<0.001
Habitat×Age	1, 124	14.87	<0.001
Habitat×Tissue	2, 121	3.33	0.039
Age×Tissue	2, 121	0.40	0.671
Habitat×Age×Tissue	2, 121	0.84	0.433
δ^15^N			
Habitat	1, 124	83.34	<0.001
Age	1, 124	9.38	0.003
Tissue	2, 121	1.36	0.261
Habitat×Age	1, 124	17.54	<0.001
Habitat×Tissue	2, 121	1.74	0.180
Age×Tissue	2, 121	2.71	0.071
Habitat×Age×Tissue	2, 121	4.01	0.021

**Table 2:** Average δ^13^C and δ^15^N values of potential prey available in coastal and inland habitats in Iceland.

Prey	Habitat	δ^13^C	δ^15^N
Black Guillemot (*Cepphus grille*)	Coastal	−17.41	14.28
Marine fish (*Myxocephalus scorpius*)	Coastal	−15.75	15.91
Starfish (Echinoderma)	Coastal	−14.47	11.42
Eider (*Somateria mollissima*)	Coastal	−19.99	8.99
Ptarmigan (*Lagopus* sp.)^1^	Coastal and Inland	−23.72	2.81
Snipe (*Gallinago gallinago*)	Coastal and Inland	−25.11	7.04
Redshank (*Tringa totanus*)	Coastal and Inland	−24.52	7.12
Wood mouse (*Apodemus sylvaticus*)	Coastal and Inland	−24.84	10.28

1 ) Data from interior Alaska [56].

The prey table is not comprehensive and data are not intended for quantitative analyses, but rather to exemplify the wider isotope niche width that is available in coastal habitats.

**Figure 3:**
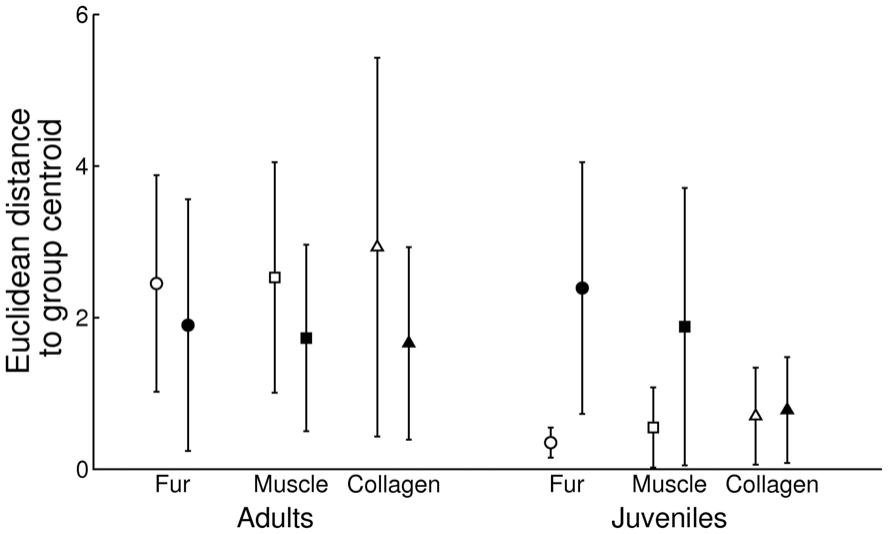
Isotope niche breadth of adult and juvenile foxes from coastal (open symbols) and inland (closed symbols) habitats on Iceland. Isotope niche breadth was estimated as the Euclidian distances to group centroids in a 2 dimensional isotope space formed by δ^13^C and δ^13^N. Figure presents mean ± 1 SE.

There was a significant interaction effect of habitat and age on isotope niche breadth (F_1, 124_ = 15.4, p<0.01) and a trend for an interaction effect of age and tissue (Table 3). Adult foxes from the coastal habitat had a broader isotope niche breadth compared to adult foxes from the inland habitat. This difference was consistent across tissues (Figure 3). Conversely, juvenile foxes from the coastal habitat had a narrower niche breadth compared to juvenile foxes from the inland habitat. Furthermore, juvenile foxes from the coastal habitat had a narrower niche breadth compared to adults, whereas there were no marked differences between adult and juvenile foxes from the inland habitat (Figure 3).

**Table 3:** Results from a linear mixed model on the effects of habitat (coastal or inland), age of animal (adult or juvenile) and tissue (fur, muscle and collagen) on the Euclidean distance to group centroids in a 2 dimensional isotope space formed by respective δ^13^C and δ^15^N values in Icelandic arctic foxes.

Fixed effect	*DF*	*F*	*P*
Habitat	1, 124	0.51	0.474
Age	1, 124	6.57	0.012
Tissue	2, 121	3.17	0.046
Habitat×Age	1, 124	15.41	<0.001
Habitat×Tissue	2, 121	1.95	0.147
Age×Tissue	2, 121	2.94	0.057
Habitat×Age×Tissue	2, 121	1.37	0.259

These distances can be interpreted as a measure of population wide isotope niche breadth.

**Figure 4:**
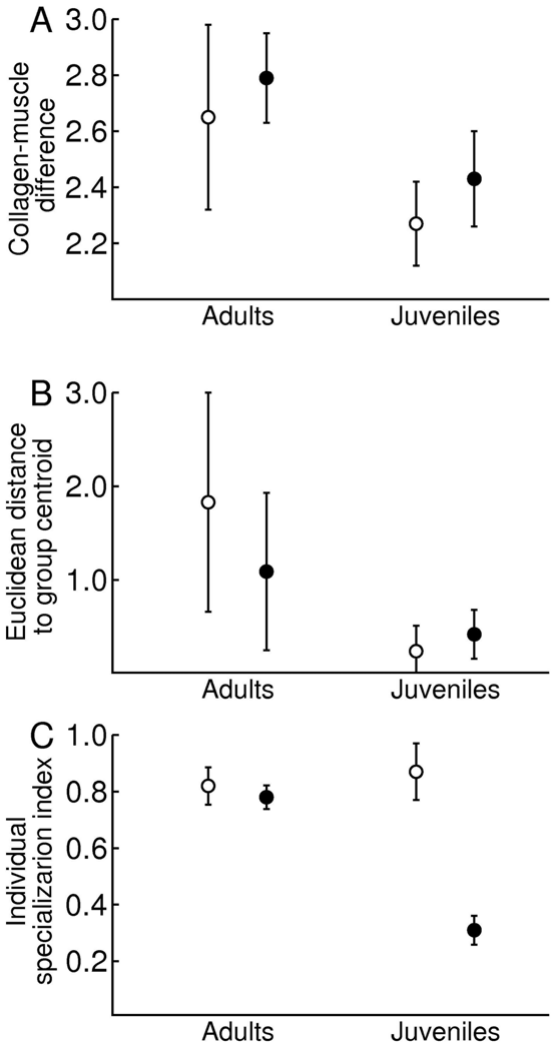
Individual isotope niche breadth (A), individual variation in individual isotope niche breadth (B) and the ratio of individual isotope niche breadth to population isotope niche breadth (C) of adult and juvenile foxes from coastal (open symbols) and inland (closed symbols) habitat on Iceland. Individual isotope niche breadth was estimated as the Euclidean distance between collagen and muscle within individuals, between individual variation in individual isotope niche breadth was calculated as the Euclidean distance of each individual difference between muscle and collagen to group centroids in a 2 dimensional isotope space, and the individual specialization index relates individual niche breadth (WIC) to the total isotope niche breadth of each sample group (TNW). We calculated this proxy for WIC/TNW (following nomenclature of Roughgarden [1]) as the ratio of the average Euclidean distances of muscle and collagen samples to within individual centroids and the average Euclidean distances to group centroids. Groups were in all cases defined as age classes within each habitat. Figures presents mean ± 1 SE.

Both habitat (F_1, 35_ = 3.89, p = 0.04) and age (F_1, 35_ = 3.95, p = 0.05) influenced individual isotope niche breadth, estimated as individual differences between muscle and collagen in δ^13^C and δ^13^N, as well as individual variation of individual isotope niche breadth (habitat: F_1, 35_ = 12.1, p<0.01; age: F_1, 35_ = 12.9, p<0.01; Table 4). Individual isotope niche breadth was higher in adults than in juveniles from both habitats (Figure 4a). Individual variation in individual isotope niche breadth was also higher in adults than in juveniles, but adult foxes from coastal habitats had higher variation than adult foxes from inland habitats (Figure 4b). There was also a significant interaction effect between habitat and age on our two tissue isotope proxy of WIC/TNW (F_1, 4_ = 11.2, p = 0.03; Table 4), with juvenile foxes from inland habitats exhibiting substantially lower individual specialization indices that both adults and juvenile foxes from coastal habitats (Figure 4c).

**Table 4:** Results from linear mixed models on the effects of habitat (coastal or inland) and age of animal (adult or juvenile) on three attributes of individual variation in δ^13^C and δ^15^N.

Fixed effect	*DF*	*F*	*P*
Within individual difference between collagen and muscle			
Habitat	1, 35	4.12	0.050
Age	1, 35	3.95	0.054
Habitat×Age	1, 4	1.21	0.334
Euclidean distances to group centroids in difference between collagen and muscle			
Habitat	1, 35	12.1	0.001
Age	1, 35	12.9	0.001
Habitat×Age	1, 4	2.24	0.209
Individual specialization index			
Habitat	1, 35	10.25	0.003
Age	1, 35	39.6	<0.001
Habitat×Age	1, 4	11.2	0.029

Within individual isotope niche breadth was estimated as the Euclidean distance between collagen and muscle within individuals, between individual variation in individual isotope niche breadth was calculated as the Euclidean distance of each individual difference between muscle and collagen to group centroids in a 2 dimensional isotope space, and an individual specialization index that relates intra individual variation to the total isotope niche breadth of each sample group, calculated as the ratio of the average Euclidean distances of muscle and collagen samples to within individual centroids and the average Euclidean distances to group centroids. Groups were in all cases defined as age classes within each habitat.

## Discussion

In this study we used stable isotope data to infer contrasting dietary strategies between arctic foxes with and without direct access to coastal habitats in Iceland. Our analyses points to three main results. First, adult foxes from coastal and inland habitats appear to have foraged from different isotope niches, with isotope values from coastal habitats being enriched in both ^13^C and ^15^N, but also reflecting a broader isotope niche space compared to inland habitats. Both of these results point to a higher incorporation of marine protein in foxes from the coastal habitat [50], [51], supporting previous isotopic [52] and direct observational [16] diet studies of Icelandic arctic foxes. This highlights the importance of coastal regions for terrestrial carnivores, since it allows them to feed on prey from multiple biomes. However, the differences in isotope ratios and isotope niche breadth were also influenced by age and tissue, which suggests temporal variation in resource use as well as variation between adults and juveniles.

Our second main result relates to individual isotope niche breadth in adults. Our study suggest that the increased prey diversity offered by coastal habitats was not fully utilized on an individual level, since the average intra individual differences between muscle and collagen was not higher in adult foxes from coastal habitats. The dietary specialization index generally supported adult arctic foxes as generalist foragers, but also did not indicate any differences in the degree of specialization relative to the total isotope niche breadth between each habitat. However, there was a larger variation between individuals in individual isotope niche breadth in adults from coastal versus inland habitats. These results suggest that the wider isotope niche breadth exhibited by adult foxes in coastal habitats was caused by a diversification of individual strategies compared to inland habitats. Some coastal individuals had a comparably broader niche while others retained a narrower niche similar to inland habitats. Coastal habitats are generally more heterogeneous than inland, with access to seabird colonies and productive coastlines varying geographically. This geographic heterogeneity in resource abundance could explain the observed results if individual strategies were dictated by the locally abundant resources. Such flexibility in dietary strategies would support previous studies on the species, since both opportunism [53] and individual dietary specialization have been suggested [52], [54].

Thirdly, the effect of habitat on individual niche breadth differed between adults and juveniles. Although we had a limited number of juveniles from coastal habitats, our results suggest that adult foxes adopted different strategies for selecting resources to consume for themselves compared to resources to bring back to feed their offspring. Juveniles from coastal habitats exhibited a narrower isotope niche compared to adults and inland juveniles, and juveniles from both habitats had lower intra individual variation than adults. Moreover, inland juveniles had a substantially lower individual specialization compared to adults and coastal juveniles. Combined, these results suggest that adults were highly selective when selecting prey to feed their offspring, but not that the degree of selectivity differed between the habitats. Instead, it seems that foxes in coastal habitats were more uniform in the resources they provided offspring, whereas there was a larger individual variation in strategies to feed offspring in inland habitats. For the red fox (*Vulpes vulpes*), certain prey types are more profitable to bring back to a den, while others are more profitable to eat while out foraging [55]. Juveniles from the coastal habitat were enriched in both ^13^C and ^15^N compared to adults, whereas this age related difference was much less prominent in the inland habitat. The observed juvenile isotope variation therefore suggests that coastal foxes seem to preferentially utilize marine resources to feed young. In coastal Iceland, adult and juvenile seabirds are an important marine resource, while migrant passerines and waders are important terrestrial prey in summer [16], and possibly even invertebrates such as bumble bees [12]. Our results thus support the observation that larger prey are more profitable to bring back to the dens to feed young, and highlight that specific components of the increased range of dietary options in coastal habitats are utilized to maximize offspring survival and subsequently reproductive success.

To conclude, many studies have suggested that coastal habitats offer a higher productivity for terrestrial predators compared to inland. Our results suggest that for a small generalist predator, the arctic fox, increased food diversity in coastal habitats resulted in fox populations with a broader isotope niche space. Furthermore, this broader niche seemed to have been caused by costal foxes adopting a wider range of individual strategies rather than by either using a broader niche or by increasing individual differences in niche use. There was a large influence of age on the effect of habitat on individual niche breadth. Juveniles generally had a narrower niche breadth than adults, but although individual niche breadth per se did not differ between habitats, there was a larger variation between juvenile individuals in the inland habitat. Our interpretation of these results is that foxes in coastal habitats preferentially utilized marine resources to feed young at the dens. On a larger scale, we argue that energy exchange between marine and terrestrial environments may be crucial for the sustainability of many carnivore populations, and that coastal habitats should be regarded as high priority areas in terms of conservation of generalist predators, since they appear to offer a wide variety of dietary options that better may allow for dietary flexibility and hence the viability of individual predators.

## Supporting Information

Table S1 Isotope values (mean ± 1 sd) and number of analyzed samples (in brackets) of Icelandic arctic foxes.
(DOC)
