# Supplementary material for: The Influence of Coastal Access on Isotope Variation in Icelandic Arctic Foxes
Source: PLoS One. 2012 Mar 1;7(3):e32071. doi: 10.1371/journal.pone.0032071 (PMC3291546; doi:10.1371/journal.pone.0032071)
Supplement: Table S1 — Isotope values (mean ± 1 sd) and number of analyzed samples (in brackets) of Icelandic arctic foxes. (DOC) [file pone.0032071.s001.doc]

|  |  |  |
| --- | --- | --- |
|  | Coastal | Inland |
|  | δ^13^C (‰) | |
| Juveniles |  |  |
| Fur | -17.65 ± 0.42 (5) | -22.10 ± 1.84 (13) |
| Muscle | -18.37 ± 0.48 (6) | -23.14 ± 1.57 (38) |
| Collagen | -16.10 ± 0.74 (6) | -22.35 ± 0.45 (5) |
| Adults |  |  |
| Fur | -20.65 ± 2.05 (22) | -22.95 ± 1.37 (59) |
| Muscle | -21.61 ± 1.99 (19) | -23.95 ± 1.24 (58) |
| Collagen | -19.00 ± 2.17 (5) | -21.35 ± 1.26 (29) |
|  | δ ^15^N (‰) | |
| Juveniles |  |  |
| Fur | 12.71 ± 0.14 (5) | 8.38 ± 2.24 (13) |
| Muscle | 13.00 ± 0.59 (6) | 7.13 ± 2.08 (38) |
| Collagen | 13.12 ± 0.64 (6) | 6.19 ± 0.96 (5) |
| Adults |  |  |
| Fur | 9.52 ± 2.02 (22) | 6.91 ± 1.88 (51) |
| Muscle | 9.78 ± 2.16 (19) | 7.18 ± 1.70 (50) |
| Collagen | 9.60 ± 3.07 (5) | 6.44 ± 1.64 (28) |
